# Identification of putative allergens from school shark, a cartilaginous food fish

**DOI:** 10.1186/2045-7022-4-S2-P4

**Published:** 2014-03-17

**Authors:** Hemashree Golla, Christiane Hilger, François Hentges, Annette Kuehn

**Affiliations:** 1 CRP-Santé, Laboratory of Immunogenetics and Allergology, Luxembourg, Luxembourg; 2 Centre Hospitalier de Luxembourg, National Unit of Immunology-Allergology, Luxembourg, Luxembourg

## Background

Fish is counted among the major allergenic foods causing mild to severe IgE-mediated reactions. Parvalbumin (PV), a highly stable muscle protein, has been described as the fish panallergen in different fishes. School shark (Galeorhinus galeus), a cartilaginous fish called 'petite roussette', is a popular food fish in the French cuisine. In this study, we aimed at identifying putative allergens from school shark.

## Methods

Proteins were purified by column chromatography. Protein sequences and identities were determined by N-terminal sequencing and mass spectrometric (MS) analysis. Isoelectric points (IP) were identified using 2D-polyacrylamide gel electrophoresis (PAGE). Protein structures were analyzed by circular dichroism (CD) spectroscopy. Antibody binding to purified proteins was determined using commercial antibodies and fish-allergic patient sera by immunoblot and ELISA.

## Results

Two PV-like proteins were purified to homogeneity from shark muscle. Edman analysis gave two N-termini of 20 residues with high homology to PV database sequences. MS analysis revealed molecular weights of 11.3 kDa and 11.9 kDa, respectively. Peptide mass fingerprint analysis resulted into high coverage (79 %) for both PV. The 11.3 kDa-PV had high identity to beta-PV (65 %) and the 11.9 kDa-PV to alpha-PV (81 %). Absorption maxima for both PV were detected at 260 nm indicating a high content of phenylalanine residues. CD analysis showed characteristic spectra of alpha-helical protein structures. Calcium-saturated PV defolded upon heating and refolded upon cooling. Melting points varied for both proteins (66.6°C and 74.1°C, respectively). The 11.3 kDa-PV protein structure turned out to be sensitive to calcium-depletion in contrast to the other PV. While the 11.3 kDa-PV migrated at ca. 6 kDa in 2D-PAGE, the 11.9 kDa-PV migrated at ca. 15 kDa. IP's were at pH 4.5 and pH 5.5, respectively. Both PV were detected by anti-PV antibodies using immunoblot and ELISA. The recognition by specific IgE-antibodies varied for both shark parvalbumins when using sera from five fish-allergic patients in ELISA.

## Conclusion

Two PV with distinct protein features (sequence, calcium binding, structural stability) were identified from school shark. The relevance of shark PV's as food allergens has to be further analyzed in fish-allergic patients.

